# Measurement of retinal nerve fiber layer thickness with a deep learning algorithm in ischemic optic neuropathy and optic neuritis

**DOI:** 10.1038/s41598-022-22135-x

**Published:** 2022-10-12

**Authors:** Ghazale Razaghi, Ehsan Hedayati, Marjaneh Hejazi, Rahele Kafieh, Melika Samadi, Robert Ritch, Prem S. Subramanian, Masoud Aghsaei Fard

**Affiliations:** 1 Medical Image Research Center, School of Advanced Technologies in Medicine, Tehran University of Medical Sciences, Tehran, Iran; 2 Farabi Eye Hospital, Tehran University of Medical Sciences, Qazvin Sq, Tehran, 13138 Iran; 3 Depatment of Engineering, Durham University, South Road, Durham, UK; 4 Einhorn Clinical Research Center, New York Eye and Ear Infirmary of Mount Sinai, New York, NY USA; 5 Departments of Ophthalmology, Neurology, and Neurosurgery, School of Medicine, University of Colorado, Aurora, CO USA

**Keywords:** Eye diseases, Biomedical engineering

## Abstract

This work aims at determining the ability of a deep learning (DL) algorithm to measure retinal nerve fiber layer (RNFL) thickness from optical coherence tomography (OCT) scans in anterior ischemic optic neuropathy (NAION) and demyelinating optic neuritis (ON). The training/validation dataset included 750 RNFL OCT B-scans. Performance of our algorithm was evaluated on 194 OCT B-scans from 70 healthy eyes, 82 scans from 28 NAION eyes, and 84 scans of 29 ON eyes. Results were compared to manual segmentation as a ground-truth and to RNFL calculations from the built-in instrument software. The Dice coefficient for the test images was 0.87. The mean average RNFL thickness using our U-Net was not different from the manually segmented best estimate and OCT machine data in control and ON eyes. In NAION eyes, while the mean average RNFL thickness using our U-Net algorithm was not different from the manual segmented value, the OCT machine data were different from the manual segmented values. In NAION eyes, the MAE of the average RNFL thickness was 1.18 ± 0.69 μm and 6.65 ± 5.37 μm in the U-Net algorithm segmentation and the conventional OCT machine data, respectively (P = 0.0001).

## Introduction

Optical coherence tomography (OCT) has been applied to measure peripapillary retinal nerve fiber layer (RNFL) thickness at a micrometer scale in several optic neuropathies such as non-arteritic anterior ischemic optic neuropathy (NAION) and demyelinating optic neuritis (ON)^1–3^. In the early stages of NAION and papillitis, OCT of the RNFL may show thickening that decreases in the subacute phase. An accurate measurement of the thickness is important as RNFL thickness may be useful for detection of disease progression or improvement^2,3^. Commercial OCT machines use automated retinal layer segmentation algorithms to detect the difference in signal intensity between adjacent retinal layers to calculate RNFL thickness. However, the scans can be affected by movement, media opacity, algorithm failure or poor signal to noise ratios and misidentification of the anterior and posterior boundaries of the RNFL and incomplete segmentation are artifacts that are consistently described in glaucoma eyes^4–6^. In some studies, the artifact rates of 46.3% and 61.7% for OCT B scans in glaucoma eyes were reported^4,7^. Such errors may cause false measurements of the thicknesses of the different layers and structures. Of note, the baseline errors persist into the subsequent scans, and errors are propagated longitudinally^8^. Therefore, manual refinement of OCT retinal layer segmentation when assessing RNFL thickness by an operator is recommended, but this process is laborious and extremely time consuming. While previous studies have not determined the segmentation issues and artifacts in other optic neuropathies such as NAION and ON, it has been reported in a case report that segmentation errors can lead to clinical misdiagnosis of neuro-ophthalmic diseases if they go unrecognized^9^.

Deep learning (DL) is a type of artificial intelligence that uses multilayer neural networks, and its algorithms outperform ophthalmologists in disease detection^10^. Furthermore, DL algorithms have been trained to detect errors in automated RNFL segmentation of OCT scans in glaucoma, identifying the probability of a segmentation artifact as well as highlighting the location of these errors using a heat-map with an accuracy of 92.4%^11^. However, studies are lacking about the use of DL for RNFL segmentation in NAION and ON eyes.

Since accurate RNFL thickness data is necessary for diagnosis and follow up of ischemic optic neuropathy and optic neuritis^10^, we examined OCT machine segmentation errors in individuals with NAION and ON and hypothesized that a DL approach with accurate segmentation of the retinal layers would allow estimation of the thickness of the RNFL which is comparable to manual segmentation. In addition, we hypothesized this approach would outperform conventional OCT machine RNFL data segmentation algorithms.

## Results

The training/validation dataset included 750 RNFL OCT B-scans from 250 eyes (60 eyes with NAION, 50 eyes with ON, and 140 control eyes). These study eyes were split into training (80% of the sample) and validation (20%) datasets.

Performance of our algorithm was evaluated in 370 scans acquired in 132 eyes from the test set. There was no overlap between the training and testing sets.

After excluding 10 scans due to centration and quality issues, the final test set consisted of 194 OCT B-scans from 70 healthy eyes, 82 scans from 28 NAION eyes, and 84 scans of 29 ON eyes. Five scans (7.1%) from healthy eyes, three scan (3.5%) from ON eyes, and 53 scan (64%) from NAION eyes had segmentation errors which were corrected manually (Chi-Square; P < 0.001). In addition, eight scans (9.7%) from NAION eyes and one scans from both ON and healthy eyes had epiretinal membrane (P < 0.001). Partially posterior vitreous detachment was seen in 20.7% (17) NAION scans and 10% (7) healthy scan and 9.5% (8) ON scans (P < 0.001). The mean of the manually corrected average RNFL thickness (ground-truth) was significantly lower in NAION (69.7 ± 19.3 μm) and ON (76.0 ± 16.2 μm) eyes compared to control (100.2 ± 10.8 μm) eyes (P < 0.001, Kruskal–Wallis). There was no difference in average RNFL thickness between ON and NAION eyes (P = 0.96). NAION scans had lower OCT quality scores than ON scans (23.7 ± 4.5 vs 25.7 ± 4, P = 0.03, Kruskal–Wallis) and control scans (26.8 ± 4, P < 0.001). In all datasets, the mean age at OCT scan was 62.8 ± 9.4 years in the NAION group, 30.8 ± 10.1 years in the ON group, and 42.3 ± 18.4 in the controls.

We conducted a two-step process to obtain our results: the first step was our U-net evaluation, and the second step was considering the RNFL thickness measurement using the U-net algorithm in the three different groups (control, ON, NAION) for the test images^12,13^.

Our U-net model yielded high performance in the test and validation images. The sensitivity and specificity of our proposed model on the validation data sets were 0.91 and 0.90, respectively. The same measures on the test sets were 0.88 and 0.86, respectively. The Dice coefficient between our proposed segmentation and manual segmentation by an expert for validation data set was 0.90, and for the test images was 0.87.

We also compared the estimate of RNFL thickness measurements in seven sectors (average, nasal, temporal, superior-temporal, superior-nasal, inferior-temporal, inferior-nasal) by three different methods (our U-Net algorithm, conventional OCT machine data, and the manual segmented best estimate determined by the ophthalmologist) in the three different groups (Table 1):

**Table 1:** Comparison of estimate of RNFL thickness measurements (µm) in seven sectors by three different methods in the control, non-arteritic anterior ischemic optic neuropathy (NAION) and demyelinating optic neuritis (ON).

Parameter	Control			P value	NAION			P value	ON			P value
	Ground truth	OCT Machine	U-Net		Ground truth	OCT Machine	U-Net		Ground truth	OCT machine	U-Net	
Average	100.3 ± 10.9	100.2 ± 11.2	100.1 ± 10.8	0.69	69.7 ± 19.3	64.4 ± 21.3	70.5 ± 19.4	0.02	76.1 ± 16.2	75.8 ± 16.6	77.1 ± 16.1	0.66
Nasal	75.6 ± 12.6	75.4 ± 12.9	76.3 ± 12.3	0.79	54.5 ± 16.9	51.3 ± 23.9	55.5 ± 16.7	0.27	57.8 ± 14.6	57.8 ± 14.5	58.9 ± 14.5	0.60
Temporal	68.4 ± 10.8	68.4 ± 10.8	68.9 ± 10.9	0.88	51.9 ± 18.2	49.6 ± 21.1	53.1 ± 18.2	0.50	47.4 ± 16.2	47.3 ± 15.9	48.3 ± 15.9	0.65
Nasal inferior	117.1 ± 22.8	116.9 ± 23.1	117.8 ± 22.9	0.92	89.9 ± 40.0	89.1 ± 42.1	90.6 ± 39.7	0.92	91.5 ± 24.4	92.6 ± 24.3	92.6 ± 24.3	0.76
Temporal inferior	145.1 ± 22.2	145.1 ± 22.2	145.8 ± 22.0	0.93	101.7 ± 43.2	100.3 ± 45.7	102.9 ± 43	0.88	108.4 ± 33.1	108.3 ± 33	109.6 ± 32.9	0.89
Nasal superior	114.8 ± 20.8	115.1 ± 20.9	114.8 ± 22.4	0.99	70.7 ± 20.7	67 ± 27.1	71.9 ± 20.6	0.40	93 ± 23.2	92.6 ± 24.1	94.1 ± 23.1	0.79
Temporal superior	137.2 ± 18.1	137.2 ± 18.1	137.8 ± 18.1	0.93	82.3 ± 34.9	77.3 ± 36.2	83 ± 35.2	0.37	105.8 ± 25.8	105.4 ± 26.7	106.7 ± 25.8	0.88

### RNFL thickness in the normal control group

**Figure 1:**
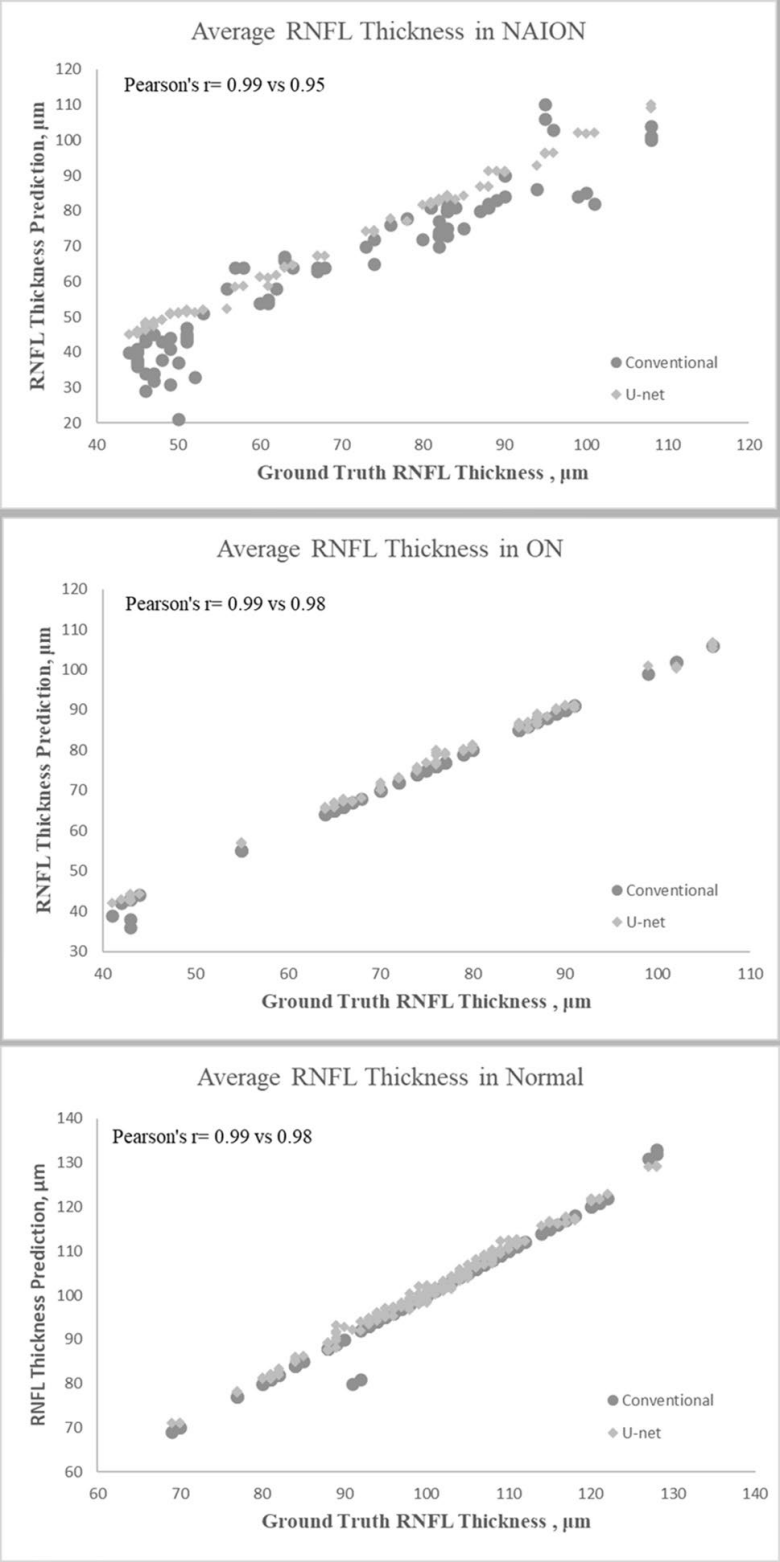
Line graphs showing correlation between average retinal nerve fiber layer (RNFL) thickness estimated by our U-Net and ground-truth in three study groups. (**A**) In anterior ischemic optic neuropathy (AION), (**B**) in optic neuritis (ON) and (**C**) in normal data.

There was no significant difference in average RNFL thickness amongst the three methods of measurements (P = 0.69, ANOVA). The mean average RNFL thickness using our U-Net algorithm segmentation was not different from the manual segmented best estimate (ground truth) (101.1 ± 10.8 µm, vs 100.2 ± 10.8 µm; P > 0.99). Similarly, there was no significant difference between the OCT machine average RNFL thickness (100.2 ± 11.2) and the manually segmented value (P > 0.99). Both RNFL thickness from U-Net algorithm segmentation and the conventional OCT machine data were strongly correlated with RNFL thickness obtained from manual segmentation (r^2^ = 0.99 and 0.98) with no significant difference between two correlations (P = 0.33) (Fig. 1). Mean absolute error (MAE) of the average RNFL thickness was 1.04 ± 0.74 μm and 0.18 ± 1.23 µm in the U-Net algorithm segmentation and the conventional OCT machine data, respectively. There was no significant difference between the two MAE numbers (P = 0.93).

### RNFL thickness in the NAION group

**Figure 2:**
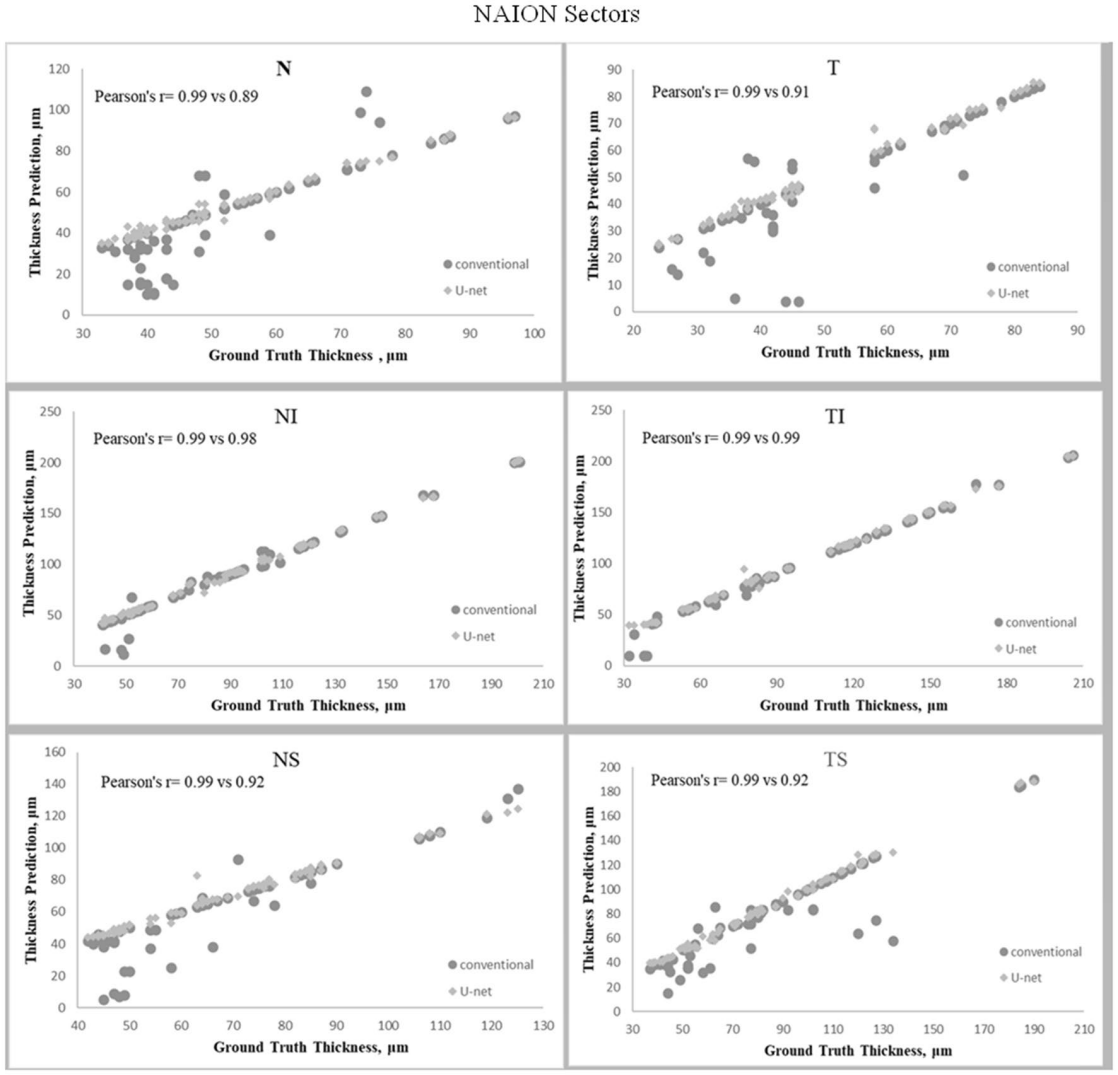
Line graphs showing correlation between each sector retinal nerve fiber layer (RNFL) thickness estimated by neural network and ground-truth in anterior ischemic optic neuropathy (AION) group. (**A**) Sector nasal (N), (**B**) sector temporal (T), (**C**) sector nasal inferior (NI), (**D**) sector temporal inferior (TI), (**E**) sector nasal superior (NS), (**F**) sector temporal superior (TS).

The Kruskal–Wallis test showed a significant difference in average RNFL thickness amongst the three methods of measurement (P = 0.02). While the mean average RNFL thickness using our U-Net algorithm was not different from the manually segmented value (ground truth) (70.5 ± 19.4 µm, vs 69.7 ± 19.3 µm, respectively; P > 0.99), the OCT machine RNFL thickness (64.4 ± 21.3 µm) was lower than the manual segmented value (P = 0.04). Furthermore, a significant difference was also found between U-Net calculated RNFL thickness and OCT machine thickness (P = 0.009). The correlation between the manually segmented RNFL thickness and the U-Net average RNFL thickness (r = 0.99) was stronger than the correlation between manually segmented RNFL thickness and the OCT machine RNFL thickness (r = 0.95) (P = 0.02) (Figs. 1, 2). The MAE of the average RNFL thickness was 1.18 ± 0.69 μm and 6.65 ± 5.37 μm in the U-Net algorithm segmentation and the conventional OCT machine data, respectively. There was a significant difference between the two MAE numbers (P = 0.0001). Specifically, the MAE for nasal, nasal superior, and temporal superior RNFL thicknesses with U-Net segmentation were 1.48 ± 1.26 μm, 1.64 ± 2.19 μm, and 1.69 ± 1.38 μm, respectively. The MAE for the corresponding thickness sectors with the conventional OCT machine were 6.16 ± 10.02 μm, 5.08 ± 10.47 μm, and 6.26 ± 13.16 μm, respectively.

### RNFL thickness in the ON group

The average RNFL thickness was not significantly different amongst the three methods of measurement (P = 0.66 Kruskal–Wallis). The mean average RNFL thickness was 76.1 ± 16.2 µm with manual segmentation, and 77.1 ± 16 µm versus 75.9 ± 16.6 µm using U-net algorithm segmentation and the OCT machine, respectively. Both average RNFL thicknesses from U-net algorithm segmentation and the conventional OCT machine data were strongly correlated with RNFL thickness obtained from manual segmentation without a significant difference between them (r = 0.99 and 0.99, respectively, P = 033) (Fig. 1). The MAE of the average RNFL thickness was 0.2 ± 1 μm and 1.2 ± 0.71 μm with OCT machine and U-Net segmentation without a significant difference between them (P = 0.93).

**Figure 3:**
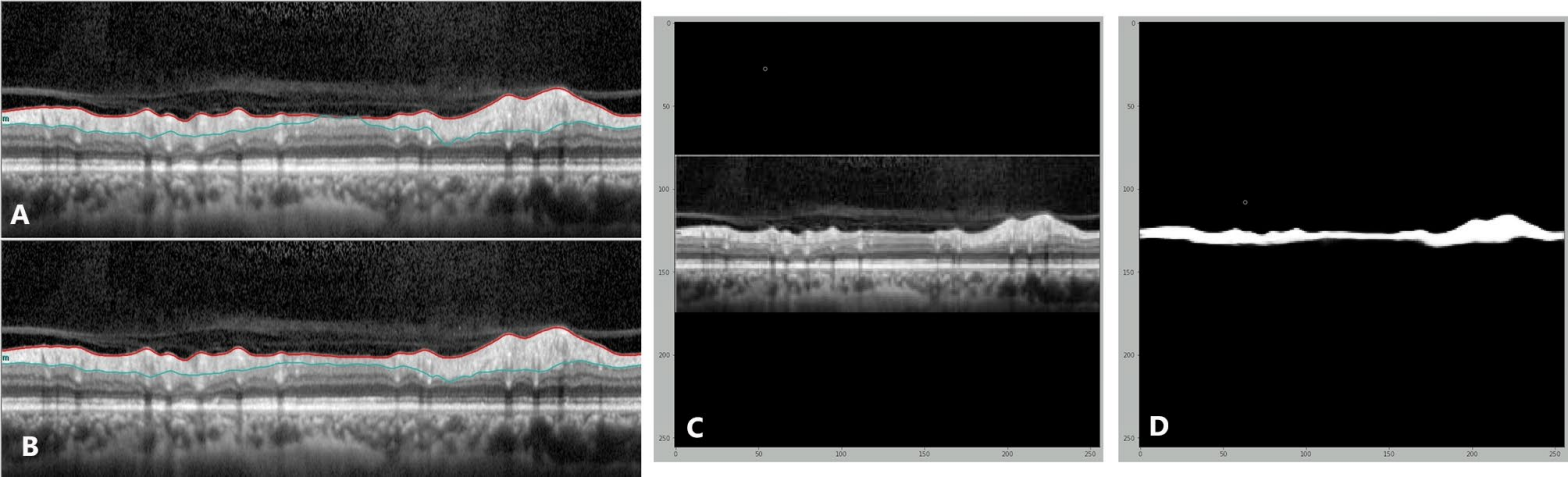
Sample OCT image of an eye with ischemic optic neuropathy. Retinal nerve fiber layer segmentation by OCT machine with error (**A**) and after manual correction (**B**). (**C**) The image input for our U-Net and (D) is the prediction mask output of our U-Net retinal nerve fiber layer segmentation, which is very similar to ground-truth (**B**).

This study investigated quantification of peripapillary RNFL thickness on OCT with deep learning (U-Net) in NAION eyes, ON eyes and controls. We compared the manually segmented (ground-truth) estimate of RNFL thickness with our U-Net algorithm and conventional OCT machine data. First, we showed high performance of our U-Net model in the test and validation images. The Dice coefficient between our model and manual segmentation for the validation data set was 0.90, and for the test images was 0.87. Second, both the RNFL thickness from U-Net algorithm segmentation and the conventional OCT machine data were similar to the RNFL thickness obtained from manual segmentation in control and ON eyes. However, in NAION eyes, the mean average RNFL thickness using the OCT machine was different from the manual segmentation (Fig. 3). In these eyes our U-Net algorithm was not different from the manually segmented value (ground truth).

Errors in segmentation of the RNFL and estimates of its thickness are not uncommon and could lead to disease misdiagnosis. Several studies have demonstrated high rates of errors on peripapillary RNFL segmentation in glaucomatous eyes.^4–7,14,15^ Mansberger et al.^5^ have found that automated OCT machine data resulted in a 1.6 μm thinner RNFL thickness than the ground-truth measurements determined by manual refinement. Manual refinement changed 8.5% of scans to a different global glaucoma classification wherein 23.7% of borderline classifications become normal. A few studies have shown RNFL segmentation problems in neuro-ophthalmology and its impact on disease follow up^9^. In this study for the first time we showed high rate of OCT machine segmentation errors in NAION eyes (64%) which causes 6.65 ± 5.37 μm mean absolute error in the mean global RNFL obtained from OCT machine automated segmentation compared to ground-truth manual segmentation. However, this error was significantly lower in ON eyes and controls. Such segmentation errors may cause false measurements of the RNFL thicknesses in post-acute NAION eyes which lead to poor prediction of patient’s vision prognosis^16,17^. Therefore, OCT machine RNFL data in NAION eyes either should be refined manually or artificial intelligence guided software must be used. The reasons for segmentation error in NAION eyes are multifactorial. Another study found three common sources of RNFL imaging artifacts: posterior vitreous detachments, high myopia, and epiretinal membranes, with the third being the most common culprit^14^. Our NAION eyes, which were older than ON and control eyes, had a higher frequency of vitreous detachment and epiretinal membrane (20.7% and 9.7% respectively). Other studies also indicated that the difference between the automated and ground-truth thickness increased with older age, thinner RNFL thickness, and lower scan quality^5^. Miki et al.^15^ also showed a 20.7% segmentation failure in glaucoma eyes, which is significantly correlated with low signal strength index and large disc area^15^. Of note, our NAION scans had lower scan quality than ON and control scans. It seems that sub-optimal scan quality reduces the accuracy of automated segmentation. A reduction in signal strength from a media opacity such as dry eye, corneal opacities, and cataract or vitreous opacities can result in artifacts in layer segmentation and interpretation^9^. Another study showed that RNFL thickness measurements from healthy and glaucoma eyes decreased as OCT scan quality decreased. They explained that more noise in lower quality images increases the likelihood that segmentation algorithms will not accurately identify boundary of RNFL layers. Increased noise also may adversely affect algorithms used for centering^18^. In the present study we found higher segmentation error in NAION eyes due to poor scan quality, epiretinal membrane and partially posterior vitreous detachment compared to ON and healthy controls.

To address the issue of OCT layer segmentations, several studies have used deep learning algorithms for retinal segmentation in normal and age-related macular degeneration eyes^19–21^. In the optic nerve head scans, Devalla et al.^22^ developed a DL algorithm which achieved good accuracy when compared to manual segmentation. The same group proposed a 3D segmentation framework (ONH-Net) that is easily translatable across OCT devices in healthy and glaucoma eyes. While they did not quantify RNFL thickness in their study, they automatically segment OCT volumes from a new OCT device without having to re-train ONH-Net with manual segmentations from that device^23^. Similar to our study Yow et al. used U-Net for RNFL segmentation and quantification with input image of cross-sectional OCT scans and generation of a binarized circumpapillary RNFL mask^24^. Furthermore, Yow et al. used the segmented RNFL from a cross-sectional OCT image and blood flow information from an enface OCT angiography image to segregate retinal vascular and neuronal components within the RNFL for thickness measurement^25^. In the view of clinical application of DL, Jammal et al.^11^ developed a DL algorithm that detects errors in RNFL segmentation in glaucoma and normal eyes. In this study, the test sample consisted of scans with at least one RNFL segmentation error and scans without error as defined by a human grader, and the algorithm was trained to output the probability of a segmentation error in test data. For a probability cut point of 0.5, the DL algorithm was 95.0% sensitive and correctly identified 1172 of the 1234 scans that had any segmentation error(s) in the test sample. The same group in another study predicted the RNFL thickness from raw unsegmented scans using DL^26^. In images without segmentation errors, they found a high correlation of segmentation-free DL RNFL predictions with conventional OCT RNFL thickness calculations. In low-quality images with segmentation errors, segmentation-free DL predictions had higher correlation with the best available estimate compared to those from the conventional OCT machine. The MAE was 4.98 ± 5.85 μm for DL RNFL estimates and 8.59 ± 11.26 μm for OCT machine estimates^26^. However, the ground truth in their study was considered the best available estimate from a good quality scan of the conventional OCT, rather than manual segmented data as a ground truth as in our work. We found MAE of the average RNFL thickness in NAION eyes with lower scan quality was 1.18 ± 0.69 μm and 6.65 ± 5.37 μm in the U-Net algorithm segmentation and the OCT machine data, respectively. Interestingly, in ON and controls, both the average RNFL thicknesses from U-net algorithm segmentation and the conventional OCT machine data were strongly correlated with RNFL thickness obtained from manual segmentation without a significant difference between them.

Our study had several limitations. First, our data set was smaller than in glaucoma studies, which is expected in light of the relative rarity of other optic neuropathies compared to glaucoma. In addition, we do not know if the segmentation performance would improve when trained upon a larger data set. Second, our U-Net was trained with the images from the Spectralis OCT machine and therefore, we could not extrapolate our algorithm to scans of other OCT devices. Third, in the absence of external validation data set in our study we could not generalized our data. Finally, our supervised DL algorithm was trained to be only as good as the manual segmentation according to an ophthalmologist which was subject to bias.

Overall, using U-Net, we were able to segment the RNFL layer in three groups of eyes. When trained and tested on compensated images, there was good correlation with manual segmentation in control eyes, ON eyes, and NAION eyes. In contrast, conventional OCT machine segmentations were prone to errors in NAION eyes, resulting in inaccurate RNFL thickness measurements. In addition, in lower quality scans, our U-Net segmentation performance was similar to ground truth, and therefore this algorithm may provide robust RNFL thickness estimates both in good quality images as well as in those that are prone to segmentation errors such as may occur in NAION eyes. Such an algorithm could be helpful in clinical practice for assessing RNFL thickness in NAION eyes as well as ON eyes.

## Material and methods

### Subjects

This was a cross-sectional comparative study in eyes with post-acute ON and NAION that were examined at Farabi Eye Hospital between May 2015 and April 2020. The study was approved by the Ethics Committee of Tehran University of Medical Sciences, and all investigations adhered to the tenets of the Declaration of Helsinki.

Informed consent was obtained from all subjects and they underwent a standard ophthalmic evaluation, including best-corrected visual acuity (BCVA), slit-lamp biomicroscopy, and SD-OCT imaging (Spectralis, HEYEX software 6.0 Heidelberg Engineering, Heidelberg, Jena, Germany) for peripapillary RNFL thickness measurement.

Diagnosis of NAION was defined by: (1) a history of sudden visual loss > 3 months prior to enrollment, with documented acute optic disc edema, (2) complete resolution of disc edema at the time of study, and (3) optic disc-related visual field defects^10^. Diagnosis of ON was defined by: (1) an attack of painful, subacute vision loss between 3 and 12 months prior to enrollment in patients age 18 to 50 years, and (2) a gadolinium-enhanced MRI demonstrating optic nerve enhancement with or without periventricular plaques typical of multiple sclerosis^27^. In both groups, patients with other ocular pathology such as glaucoma and giant cell arteritis or a history of other autoimmune diseases were excluded.

### Spectral-domain optical coherence tomography

Images of the peripapillary RNFL were acquired using the Spectralis SD-OCT, and two to four images for each study eye were used. Images with quality of less than 10 were not included. Segmentation lines were delineated by the conventional OCT software in the Spectralis SD-OCT, including the anterior and posterior RNFL boundaries in the circle scan corresponded to the internal limiting membrane and the inner plexiform layer, respectively. The peripapillary RNFL measurements thus delineated by the OCT instrument were recorded. All OCT machine segmentations were inspected by an ophthalmologist (E.H) for possible segmentation errors, manual corrections of segmentation were made to the B-scans, and subsequently RNFL thickness data from corrected segmentations were recorded as “ground-truth” data.

### Image processing and deep learning algorithm

We developed an algorithm for estimating RNFL thickness based on deep learning. At the first step, a preprocessing method was used to reduce speckle noise. After that, a segmentation algorithm based on a U-Net algorithm was implemented to delineate the contours of the RNFL. In a post-processing step, to eliminate any speckled patterns in the segmentation results, a morphological method was applied. Finally, RNFL thickness was determined using a pipeline of image processing methods and the deep learning model.

### Pre-processing

**Figure 4:**
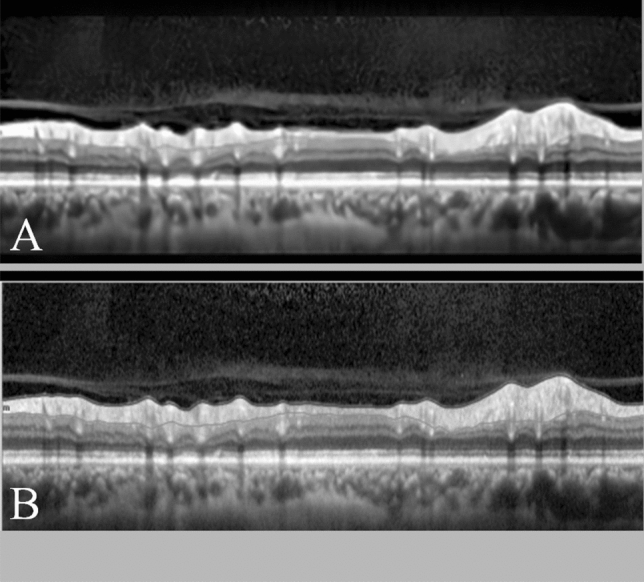
Image processing before (**A**) and after (**B**) denoising.

Speckle noise was reduced as the primary objective of the preprocessing step (Fig. 4). To minimize these image artifacts, morphological filters (using OpenCV version 4.5.1, kernel dimension: square; size: 3 × 3) were used.

### Retinal nerve fiber layer segmentation using U-Net

**Figure 5:**
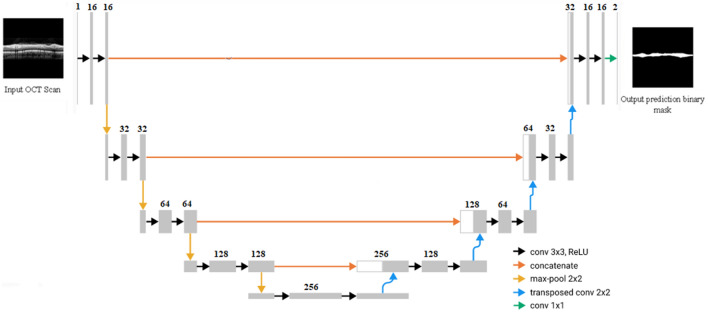
U-Net architecture overview. Input X pass forward the network and prediction mask is created by network.

In this study, the U-Net is adopted for segmentation of the RNFL. U-Net is an accurate architecture developed by Ronneberger et al.^12^ (a detailed architecture is shown in Fig. 5). To train the network and make predictions on test images, images were resized to 256 × 256 pixels by zero padding. The input image was passed to the network, and at the last convolution layer a binary mask was produced by the network that included the RNFL region. There are skip connections between encoders and decoders in the U-Net architecture. Max-pooling is used to decrease the input size by a factor of two and to capture contextual information at different resolution levels. The resolution is restored to the original level in the decoder module by up-sampling using Up-Convolution, allowing for precise localization. Moreover, the input images are passed through a series of convolutional layers with the ReLU activation function. The designed U-net utilized increasing numbers of convolutional filters (16, 32, 64, 128, 256) in the encoder and the symmetric structure in the decoder. Binary cross entropy was used as a loss function for our model. The deep learning model was implemented with Python programming language (Python 3.5 Software Foundation, https://www.python.org/). After training and validating the U-Net model using RNFL OCT images and their ground truth, we predicted the output of the unlabeled RNFL images in the test set.

### Post-processing and average RNFL thickness estimation

After implementing the segmentation method, the resulting binary masks may contain gaps and speckles. The gaps were filled by implementing post-processing methods based on a morphological algorithm. After the post-processing step, the average thickness of RNFL was measured by using a Euclidean distance transform method^13^.

### Performance metrics

We used specificity, sensitivity, and the Dice coefficient to evaluate the segmentation accuracy of our U-Net on the test sets. Specificity was used to assess the true negative rate of the proposed method and sensitivity was used to assess the true positive rate of the proposed method compared to the corresponding manually segmented images. The Dice coefficient, which measures the overlap between the manual and U-Net segmentation, is between 0 and 1, where 0 represents no overlap and 1 represents a complete overlap.

Finally, after calculating the RNFL thickness, mean absolute error (MAE) was reported to compare both the DL algorithmic RNFL results and the OCT machine RNFL data with the ground-truth, defined as the best available estimation of RNFL average thickness that the ophthalmologist determined manually.

### Statistics

The normality assumption was verified using the Shapiro–Wilk test. Normal data were analyzed by one-way analysis of variance (ANOVA), and a Kruskal–Wallis test was used when we did not assume a normal distribution of the data. A Bonferroni correction was used to account for multiple comparisons. The correlations between data were analyzed with the Pearson test, and the differences between the correlations was evaluated through the package “cocor” in R (http://www.R-project.org/; provided in the public domain by the R Foundation for Statistical Computing, Vienna, Austria). Differences were considered significant if the *P* value was less than 0.05.

## Data Availability

The datasets generated during and/or analysed during the current study are available from the corresponding author on reasonable request.
